# Abstaining from annual health check-ups is a predictor of advanced cancer diagnosis: a retrospective cohort study

**DOI:** 10.1265/ehpm.21-00292

**Published:** 2022-02-19

**Authors:** Yuki Kuwabara, Maya Fujii, Aya Kinjo, Yoneatsu Osaki

**Affiliations:** Division of Environmental and Preventive Medicine, Faculty of Medicine, Tottori University, Tottori, Japan

**Keywords:** Cancer prevention, Health check-ups, Cancer screening, Health service utilisation

## Abstract

**Background:**

Cancer prevention is a crucial challenge in preventive medicine. Several studies have suggested that voluntary health check-ups and recommendations from health professionals are associated with increased participation in cancer screening. In Japan, it is recommended that individuals aged 40–74 years should undergo annual health check-ups; however, the compliance to this recommendation is approximately <50%. According to the national survey, individuals who do not undergo annual health check-ups are at a higher risk for cancer. However, to the best of our knowledge, no previous study has investigated the association between the use of health check-ups and the incidence rate of cancer. We hypothesised that not undergoing periodic health check-ups and/or less use of outpatient medical services are predictors for advanced cancer.

**Methods:**

To explore the relationship between health check-up or outpatient service utilisation and cancer incidence, this retrospective cohort study used data at two time points—baseline in 2014 and endpoint in 2017—from the National Health Insurance (NHI) claims and cancer registry. A multivariable logistic regression analysis was performed to investigate whether cancer diagnosis was associated with health check-up or outpatient service utilisation.

**Results:**

A total of 72,171 participants were included in the analysis. The results of the multivariable logistic regression showed that individuals who skipped health check-ups had a higher risk of cancer diagnosis (odds ratio [OR], 1.21; 95% confidence interval [CI], 1.04–1.40). Moreover, not undergoing health check-ups increased the risk of advanced-stage cancer (OR, 1.78; 95% CI, 1.29–2.44). Furthermore, increased rate of outpatient service utilisation was negatively associated with advanced cancer diagnosis.

**Conclusions:**

This is the first study reporting that not undergoing health check-ups is a predictor of cancer diagnosis and advanced cancer stage. Primary prevention strategies for NHI members who do not undergo health check-ups must be reassessed. Moreover, future research should examine secondary prevention strategies, such as health education and recommendations from health professionals to facilitate adequate utilisation of preventive health services.

**Supplementary information:**

The online version contains supplementary material available at https://doi.org/10.1265/ehpm.21-00292.

## Background

Cancer is a leading cause of death in high-income countries; the national cancer registry of Japan reported that the total incidence of cancer in 2017 was 977,393 (558,869 male and 418,510 female) [1]. These numbers indicate that more than half of the Japanese population suffers from cancer at least once in their lifetime. As such, cancer prevention is a critical public health issue. To address this concern, the National Health Promotion created the Healthy Japan 21 Program and Cancer Control Act, establishing targets that aim to reduce age-adjusted cancer mortality rates in those below the age of 75 years [2].

Primary and secondary prevention efforts are traditional strategies for preventing the detrimental consequences of cancer [3]. Primary prevention aims to decrease the incidence of cancer, and secondary prevention aims to reduce mortality by promoting early cancer detection through adequate cancer screening. Previous studies suggest that secondary prevention strategies, including establishing continuous relationships with providers such as general practitioners [4–6], receiving recommendations from healthcare professionals, undergoing regular check-ups [7], increasing the frequency of visits to physicians [8, 9], and increasing contacts with healthcare professionals [10], were associated with increased cancer screening utilisation.

In the United States (US), healthcare is privately funded, and factors such as insurance type and level of coverage influence cancer screening in individuals [11–15]. However, Japan’s health insurance system is part of a universal healthcare system. Every resident is required to enrol in the employee health insurance (EHI) or National Health Insurance (NHI) [16]. The EHI covers employed individuals and their dependents, while the NHI covers sole proprietors, part-time workers, and unemployed individuals [17, 18]. The NHI is based on residence, thus local municipalities act as insurers.

Employee insurers above 40 years old are obligated to undergo ‘specific health check-ups’ at least once annually. Such check-ups involve screening for conditions, such as diabetes, hyperlipidaemia, and other non-communicable diseases. Similar specific check-ups are offered to residents enrolled in the NHI by local municipalities. However, unlike employees’ specific health check-ups, the NHI’s specific health check-ups are not compulsory. Regardless of the type of insurance, cancer screening is often optional for individuals who undergo specific health check-ups.

Although the cost of these health check-up visits is generally covered by companies or local municipalities, participation in health check-ups and cancer screening remains suboptimal. For instance, the implementation rate of specific health check-ups among the overall eligible population was approximately 50% in 2014 [19]. Additionally, breast and cervical cancer screening rates are lower in Japan than in similar high-income countries [20]. Notably, previous reports indicate that the implementation rates of health check-ups and cancer screenings are lower among NHI members than among those insured by the EHI [21]. Implementation of secondary prevention strategies is a crucial challenge for NHI members. By default, specific health check-ups do not include cancer screening; however, it is possible that these check-ups can promote earlier cancer screening, due to increased contact with health professionals [7, 10].

Primary prevention strategies focus mainly on lifestyle risk factors, such as smoking and drinking. The Japan National Health and Nutrition Survey suggested that the proportions of smokers, drinkers, and low-income households were higher among individuals who did not undergo health check-ups than among those who did [22]. Hence, it is possible that the cancer incidence among individuals who do not undergo regular health check-ups may be increased. However, there has never been a formal investigation on whether health check-ups or medical services are associated with decreased cancer morbidity under the unique healthcare and medical examination system in Japan.

The present study explored whether abstinence from health check-ups or frequency of outpatient service utilisation are predictors of cancer incidence, through a retroactive analysis of NHI and cancer registry data. We hypothesised that individuals who abstain from annual health check-ups would have an increased risk of cancer incidence. Moreover, we hypothesised that abstaining from health check-ups and/or outpatient service utilisation are predictors of advanced cancer diagnosis.

## Methods

### Study population and data collection

This retrospective cohort study used the NHI claims and cancer registry data, obtained from Tottori Prefecture, Japan, at two time points—baseline in 2014 and endpoint in 2017. Individuals who were enrolled in the NHI of Tottori Prefecture in 2014–2017 were included in the study. Since health check-ups target individuals aged 40–74 years, NHI members from that age group were selected as study participants. Individuals residing outside Tottori, as well as individuals who died during the study period were not included. The method used to combine these datasets was developed by the administrative agency in Tottori Prefecture; the cancer registry contains information on the entire population of Tottori Prefecture, while the NHI claims data concerns only NHI participants (about a quarter of the entire population). Therefore, the accuracy rate of the matching method was examined [see Additional file 1]. When examined using data from the Medical Care System for the elderly (≥75 years old), which includes the entire population of this age group regardless of the insurance provider, 96.9% of cancer cases were identified using this method.

### Measures

Outcome variables were positive cancer diagnosis and advanced cancer diagnosis, both of which were reported in the National Cancer Registry in Tottori in 2017. In addition, cancer site (e.g., topography) was included in the data. Cases were categorised according to the topography code of the International Classification of Diseases for Oncology Third Edition (ICD-O-3), for example, stomach (C16.x), colorectal (C18.x, C19.x, C20.x), lung (C34.x), prostate (C61.x), and breast (C50.x). Cancer stage at diagnosis was classified according to the definition of the Japan National Cancer Centre, which is based on the staging manual of the Surveillance Epidemiology and End Results (SEER) Program in the US. We categorised the patients as follows: early stage, *in situ* and localised only; late stage, regional by direct extension only; regional lymph node(s) involved only; and distant site(s)/lymph node(s) involved.

Predictors of interest were the history of specific health check-ups and outpatient service utilisation in 2014. NHI claims data included data on whether each insured person underwent or abstained from annual specific health check-ups. Outpatient service utilisation was defined as the number of months in which insurance claims for outpatient medical services occurred in 2014. We created categories (quartiles) of outpatient service utilisation: 0–1, 2–6, 7–10, or 11–12, based on the distribution and a previous study [9]. Potential confounders were sex, age, residential area, and the occurrence of inpatient service insurance claims in 2014. We categorised the residential municipalities into urban or rural groups according to population, population density, and total expense of annual insurance claims in 2014.

### Statistical analysis

First, a descriptive analysis of the baseline characteristics of the study participants was performed. Second, we examined the relationship between cancer diagnosis and each predictor and potential confounder using univariate logistic regression analysis. In addition, the association between cancer diagnosis and the history of specific health check-ups and outpatient service utilisation was examined using multivariable logistic regression analysis. Potential confounders in the multivariate model were selected after consideration of data availability, adequacy, model fitting, and multicollinearity. Likewise, we excluded participants who were diagnosed with early stage, unknown, or not applicable cancer, and univariate and multivariate logistic analyses were conducted to examine the association between the outcome variable (advanced cancer diagnosis) and predictor variables. Lastly, among participants who were diagnosed with cancer, we conducted logistic regression analysis to examine the association between advanced cancer diagnosis and predictor variables. Patients with tumours classified as unknown stage or stage not applicable were excluded from the analysis. Moreover, supplemental multivariable logistic analysis was performed to examine the relationship between cancer diagnosis and advanced cancer diagnosis in specific regions. The most common cancers (stomach, colorectal, lung, and breast cancers) were investigated. All analyses were performed using SPSS 25.0 (IBM Corp, New York, NY, USA). Cases with missing values were not included in the analysis.

### Ethical statement

This survey was reviewed and approved by the Ethics Review Committee of Tottori University School of Medicine (approval no. 20A129). The data were anonymised prior to analysis. Because of the retrospective nature of the study, the Ethics Committee waived the need to obtain informed consent from the participants. Adequate information on the purpose and methods of the study can be found on the web page of Tottori University Hospital for potential subjects. Instructions for individuals who did not want to participate in the study are also available there, stating that subjects could freely refuse to participate for any reason.

## Results

**Fig. 1:**
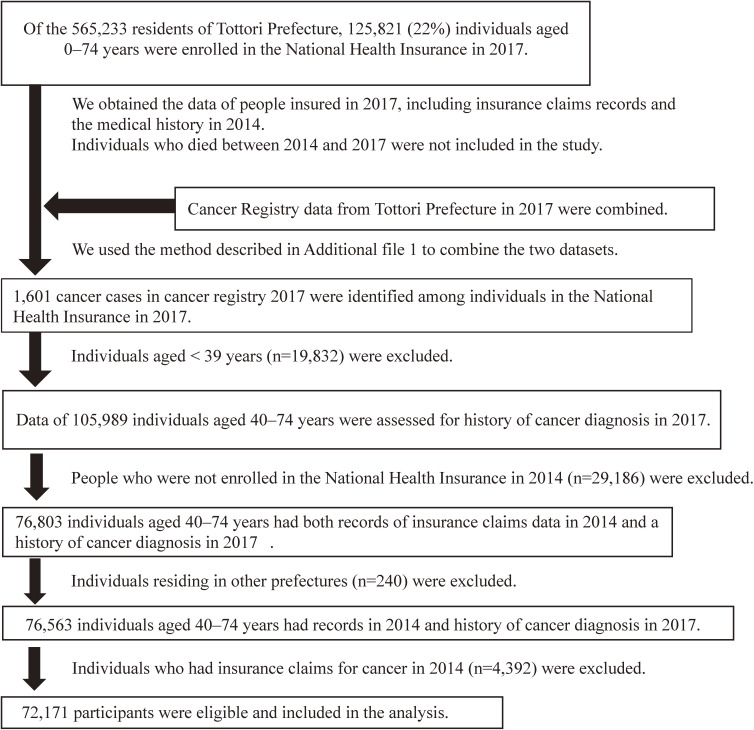
Patient selection flow chart

The flowchart of the study participants is shown in Fig. 1. Of the overall population of Tottori Prefecture in 2017 (565,233), NHI members were 125,821 (22%). A total of 72,171 participants were included in the analysis after screening for eligibility.

Table 1 shows the baseline characteristics of the study participants. A total of 920 cancer cases were identified in 2017. Of all the participants, 27.7% underwent a specific health check-up, and 68.2% claimed outpatient medical services for more than two months of the year. The number of cases and stage of the cancers identified in this study are shown in Additional file 2. The baseline characteristics of participants according to health check-up participation are shown in Additional file 3.

**Table 1:** Baseline data of study participants

	**Participants who were diagnosed with cancer in 2017**		**Participants who were not diagnosed with cancer in 2017**		**Total**	
	**n = 920, 1.3%**		**n = 71251, 98.7%**		**n = 72171**	

	**n**	**(%)**	**n**	**(%)**	**n**	**(%)**
Sex						
Female	390	(42.4)	38371	(53.9)	38761	(53.7)
Male	530	(57.6)	32880	(46.1)	33410	(46.3)
Age						
40–49 years	23	(2.5)	8859	(12.4)	8882	(12.3)
50–59 years	68	(7.4)	9665	(13.6)	9733	(13.5)
60–69 years	455	(49.5)	32647	(45.8)	33102	(45.9)
70–74 years	374	(40.7)	20080	(28.2)	20454	(28.3)
Residential area						
Urban	549	(59.7)	42087	(59.1)	42636	(59.1)
Rural	371	(40.3)	29164	(40.9)	29535	(40.9)
Health check-up in 2014						
Received	251	(27.3)	19765	(27.7)	20016	(27.7)
Abstained	669	(72.7)	51486	(72.3)	52155	(72.3)
Number of months of insurance claims for outpatient medical services in 2014						
0–1	244	(26.5)	22680	(31.8)	22924	(31.8)
2–6	212	(23.0)	17742	(24.9)	17954	(24.9)
7–10	187	(20.3)	12031	(16.9)	12218	(16.9)
11–12	277	(30.1)	18798	(26.4)	19075	(26.4)
Insurance claims for inpatient medical services in 2014						
None	845	(91.8)	66405	(93.2)	67250	(93.2)
At least once	75	(8.2)	4846	(6.8)	4921	(6.8)

Table 2 shows the results of the logistic regression analysis of the predictors of any cancer diagnosis. Although abstaining from health check-ups was not significantly associated with cancer diagnosis in the univariate model (odds ratio [OR], 1.02; 95% confidence interval [CI], 0.88–1.18; P = 0.78), the relationship was significant when adjusted for sex, age, and residential area (model 1: OR, 1.18; 95% CI, 1.02–1.37; P = 0.03). After considering outpatient service utilisation and the occurrence of inpatient service insurance claims (Model 3), the association was still significant (OR, 1.21; 95% CI, 1.04–1.40; P = 0.02). This indicates that cancer patients underwent health check-ups less frequently than control group participants.

**Table 2:** Association between predictors and any cancer diagnosis (N = 72,171; 920 cancer cases)

		**Unadjusted**			**Multivariable Model 1^a^**			**Multivariable Model 2^b^**			**Multivariable Model 3^c^**		

	**n**	**OR**	**(95% CI)**	**p-value**	**OR**	**(95% CI)**	**p-value**	**OR**	**(95% CI)**	**p-value**	**OR**	**(95% CI)**	**p-value**
Health check-up in 2014													
Received	20016	1.00			1.00			NA			1.00		
Abstained	52155	1.02	(0.88, 1.18)	0.78	1.18	(1.02, 1.37)	0.03	NA		NA	1.21	(1.04, 1.40)	0.02
Number of months of insurance claims for outpatient medical services in 2014													
0–1	22924	1.00			NA			1.00			1.00		
2–6	17954	1.11	(0.92, 1.34)	0.27	NA		NA	1.03	(0.86, 1.25)	0.73	1.07	(0.88, 1.29)	0.50
7–10	12218	1.45	(1.19, 1.75)	<0.01	NA		NA	1.17	(0.96, 1.42)	0.12	1.21	(0.99, 1.47)	0.07
11–12	19075	1.37	(1.15, 1.63)	<0.01	NA		NA	1.07	(0.89, 1.28)	0.48	1.10	(0.92, 1.32)	0.29
Sex													
Female	38761	1.00			1.00			1.00			1.00		
Male	33410	1.59	(1.39, 1.81)	<0.01	1.81	(1.59, 2.07)	<0.01	1.85	(1.62, 2.12)	<0.01	1.83	(1.60, 2.09)	<0.01
Age		1.07	(1.06, 1.08)	<0.01	1.08	(1.07, 1.09)	<0.01	1.07	(1.06, 1.09)	<0.01	1.08	(1.06, 1.09)	<0.01
Residential area													
Urban	42636	1.00			1.00			1.00			1.00		
Rural	29535	0.98	(0.85, 1.11)	0.71	0.94	(0.82, 1.08)	0.38	0.93	(0.81, 1.06)	0.27	0.94	(0.82, 1.08)	0.38
Insurance claims for inpatient medical services in 2014													
None	67250	1.00			NA			NA			1.00		
At least once	4921	1.22	(0.96, 1.54)	0.11	NA		NA	NA		NA	1.04	(0.81, 1.32)	0.78

^a^Adjusted for health check-up in 2014, sex, age, and residential area

^b^Adjusted for number of months of insurance claims for outpatient medical services in 2014, sex, age, and residential area

^c^Adjusted for health check-up in 2014; number of months of insurance claims for outpatient medical services in 2014; sex; age; residential area; and insurance claims for inpatient medical services in 2014

N: number of participants included in the analysis. n: number of participants categorized into a group.

The associations between advanced cancer diagnosis and the predictors are presented in Table 3. Regardless of the adjustment for potential covariates, abstaining from health check-ups was a significant predictor of advanced cancer diagnosis.

**Table 3:** Association between predictors and advanced cancer diagnosis (N = 71,658; 407 cancer cases)

			**Unadjusted**			**Multivariable Model 1^a^**			**Multivariable Model 2^b^**			**Multivariable Model 3^c^**		

	**n**	**Percentage of cancer cases**	**OR**	**(95% CI)**	**p-value**	**OR**	**(95% CI)**	**p-value**	**OR**	**(95% CI)**	**p-value**	**OR**	**(95% CI)**	**p-value**
Health check-up in 2014														
Received	19850	20.9	1.00			1.00			NA			1.00		
Abstained	51808	79.1	1.45	(1.14, 1.85)	<0.01	1.68	(1.32, 2.15)	<0.01	NA		NA	1.66	(1.29, 2.12)	<0.01
Number of months of insurance claims for outpatient medical services in 2014														
0–1	22811	32.2	1.00			NA			1.00			1.00		
2–6	17839	23.8	0.68	(0.73, 1.23)	0.68	NA		NA	0.88	(0.68, 1.15)	0.35	0.97	(0.74, 1.26)	0.79
7–10	12100	17.0	0.96	(0.74, 1.33)	0.96	NA		NA	0.80	(0.59, 1.08)	0.14	0.89	(0.66, 1.20)	0.43
11–12	18908	27.0	0.92	(0.79, 1.31)	0.92	NA		NA	0.78	(0.60, 1.02)	0.07	0.87	(0.67, 1.14)	0.31
Sex														
Female	38535	40.3	1.00			1.00			1.00			1.00		
Male	33123	59.7	1.73	(1.42, 2.11)	<0.01	1.93	(1.58, 2.36)	<0.01	1.97	(1.61, 2.41)	<0.01	1.92	(1.57, 2.35)	<0.01
Age			1.07	(1.05, 1.08)	<0.01	1.08	(1.06, 1.10)	<0.01	1.08	(1.06, 1.10)	<0.01	1.08	(1.07, 1.10)	<0.01
Residential area														
Urban	42329	59.5	1.00			1.00			1.00			1.00		
Rural	29329	40.5	0.98	(0.81, 1.20)	0.87	0.97	(0.80, 1.19)	0.79	0.94	(0.77, 1.14)	0.52	0.98	(0.80, 1.19)	0.81
Insurance claims for inpatient medical services in 2014														
None	66786	93.6	1.00			NA			NA			1.00		
At least once	4872	6.4	0.94	(0.63, 1.39)	0.74	NA		NA	NA		NA	0.83	(0.55, 1.24)	0.36

^a^Adjusted for health check-up in 2014, sex, age, and residential area

^b^Adjusted for number of months for which insurance claims for outpatient medical service were applicable in 2014, sex, age, and residential area

^c^Adjusted for health check-up in 2014, number of months of insurance claims for outpatient medical services in 2014; sex, age, residential area, and insurance claims for inpatient medical services in 2014

N: number of participants included in the analysis. n: number of participants categorized into a group.

The risk of advanced cancer diagnosis was also assessed among individuals who were diagnosed with any cancer (Table 4). Abstaining from health check-ups increased the risk of advanced-stage cancer diagnosis (unadjusted OR, 1.91; 95% CI, 1.40–2.59; P < 0.01). After adjustment, the association was consistently significant (Model 3: OR, 1.78; 95% CI, 1.29–2.44; P < 0.01). Furthermore, outpatient service utilisation was significantly associated with risk of cancer diagnosis in the analysis: participants who claimed outpatient service utilisation for more than seven months of the year were less likely to be diagnosed with advanced-stage cancer than those who had insurance claims for less than one month.

**Table 4:** Risk of advanced cancer diagnosis among individuals who were diagnosed with any cancer in 2017 (N = 882; 407 advanced cancer cases)

			**Unadjusted**			**Multivariable Model 1^a^**			**Multivariable Model 2^b^**			**Multivariable Model 3^c^**		

	**n**	**Percentage of cancer cases**	**OR**	**(95% CI)**	**p-value**	**OR**	**(95% CI)**	**p-value**	**OR**	**(95% CI)**	**p-value**	**OR**	**(95% CI)**	**p-value**
Health check-up in 2014														
Received	244	20.9	1.00			1.00			NA			1.00		
Abstained	638	79.1	1.91	(1.40, 2.59)	<0.01	1.90	(1.40, 2.59)	<0.01	NA		NA	1.78	(1.29, 2.44)	<0.01
Number of months of insurance claims for outpatient medical services in 2014														
0–1	230	32.2	1.00			NA			1.00			1.00		
2–6	206	23.8	0.67	(0.46, 0.98)	0.04	NA		NA	0.67	(0.46, 0.98)	0.04	0.73	(0.50, 1.08)	0.12
7–10	176	17.0	0.49	(0.33, 0.73)	<0.01	NA		NA	0.48	(0.32, 0.71)	<0.01	0.52	(0.34, 0.79)	<0.01
11–12	270	27.0	0.52	(0.36, 0.74)	<0.01	NA		NA	0.50	(0.35, 0.73)	<0.01	0.59	(0.40, 0.86)	0.01
Sex														
Female	373	40.3	1.00			1.00			1.00			1.00		
Male	509	59.7	1.16	(0.89, 1.52)	0.27	1.11	(0.84, 1.46)	0.47	1.15	(0.88, 1.51)	0.32	1.11	(0.84, 1.46)	0.48
Age			1.00	(0.98, 1.02)	0.98	1.00	(0.98, 1.03)	0.72	1.01	(0.99, 1.03)	0.45	1.01	(0.99, 1.04)	0.30
Residential area														
Urban	522	59.5	1.00			1.00			1.00			1.00		
Rural	360	40.5	0.98	(0.75, 1.28)	0.88	1.00	(0.76, 1.32)	0.99	0.96	(0.73, 1.27)	0.79	1.00	(0.76, 1.31)	0.98
Insurance claims for inpatient medical services in 2014														
None	812	93.6	1.00			NA			NA			1.00		
At least once	70	6.4	0.67	(0.40, 1.11)	0.12	NA		NA	NA		NA	0.72	(0.43, 1.21)	0.22

^a^Adjusted for health check-up in 2014, sex, age, and residential area

^b^Adjusted for number of months of insurance claims for outpatient medical services in 2014, sex, age, and residential area

^c^Adjusted for health check-up in 2014, number of months of insurance claims for outpatient medical services in 2014, sex, age, residential area, and insurance claims for inpatient medical services in 2014

N: number of participants included in the analysis. n: number of participants categorized into a group.

Lastly, a supplemental analysis was performed for the four most common cancers [see Additional files 4 and 5]. Colorectal cancer patients were more likely to abstain from health check-ups than control group participants [see Additional file 4], and abstaining from health check-ups increased the risk of advanced-stage stomach cancer and colorectal cancer diagnosis [see Additional file 5].

## Discussion

The results of this study show that 17.6% of all cancer patients in Tottori Prefecture in 2017 did not use outpatient medical services at all in 2014. A total of 72.7% of the cancer patients did not undergo any health check-ups in 2014. The proportion of individuals who neither underwent health check-ups nor received outpatient medical services in 2014 was 17.8% of all cancer cases reported in 2017.

These results indicate that individuals who did not undergo health check-ups had a higher risk of cancer diagnosis (Table 2). Individuals who undergo annual health check-ups are more likely to undergo cancer screening [7, 23]. In theory, it is possible that cancer is detected more frequently among those who undergo health check-ups than among those who skip health check-ups. Therefore, we believe that the cancer incidence in our study was not overestimated. The differences in lifestyle risk factors between the two groups may explain our results. A previous survey suggested that the proportions of smokers, drinkers, and those with unhealthy diets were higher among people who did not undergo health check-ups than among those who did [22, 24–26]. It is possible that cancer incidence is higher among individuals who abstain from health check-ups. Future studies should investigate the baseline health data of the group that does not undergo regular health check-ups, to discuss primary and secondary prevention strategies for this group.

Abstaining from health check-ups was a significant predictor of advanced cancer diagnosis (Tables 3 and 4). This result can be explained by the difference in health behaviour, that is, individuals who undergo annual health check-ups tend to undergo cancer screening more regularly [7, 23]. A previous large-scale population-based cohort study in Japan examined the association between colorectal cancer screening and advanced cancer diagnosis [27]. The study indicated that there was a reduction in the risk of advanced colorectal cancer diagnosis by approximately 60% in the screened group, compared with that in the unscreened group. Although the outcomes and predictors were different, the estimated risk of advanced cancer diagnosis due to abstaining from regular health check-ups in this study was comparable with results of the previous study. In addition, the frequency of outpatient medical service use was negatively associated with advanced cancer diagnosis. This finding suggests that a certain level of contact with health professionals can lead patients to adopt adequate screening behaviour. Previous studies support this finding [9]. Health professionals’ recommendations can play an important role in achieving adequate cancer screening [7, 10, 28]. Several studies have suggested that lack of insurance coverage may be one of the most important barriers to cancer screening in the US healthcare system [29]. In contrast, in Japan, policymakers have been facilitating the provision of specific health check-ups and cancer screening tests at an affordable price for all individuals under the universal healthcare system. Although the association between health literacy and cancer screening is controversial [30], the results of our study indicate that the use of medical services by asymptomatic individuals may be a key for adequate preventive health behaviour. A previous study of the Japanese population indicated that improving health literacy can be effective in encouraging preventive healthcare utilisation, particularly for NHI members who are not subject to compulsory preventive healthcare [31]. Resource allocation for educating NHI members or providing timely contact with healthcare services may be reasonable.

This study had several limitations. First, generalizability is limited, as the study participants were members of the NHI in Tottori Prefecture in Japan. However, to the best of our knowledge, this is the first study to clarify the challenges involved in cancer prevention in a population by examining the relationship between preventive health behaviour and risk of cancer diagnosis. Second, some cancer cases were probably not identified in our study. The incidence of some cancers, such as thyroid or pancreatic cancer, might be underestimated, as it is believed that they cannot be diagnosed based on physical examination alone. Additionally, cancer screenings are only recommended for certain cancer types. A previous study has also reported the difficulty of calculating cancer incidence using insurance claims data alone [32]. However, our study overcame this challenge by combining the cancer registry data and NHI claims data. In addition, we examined the accuracy of this method. Nevertheless, unlike the Medical Care System for the elderly aged ≥ 75 years, fewer people in the 40–74 years age group are enrolled in the NHI. Thus, it is unlikely that registered cancer patients are being matched with an accuracy as high as shown in Additional file 1. The objectivity of the variables in this study was a strength. Third, we could not exclude people with cancer at baseline completely. This might have affected our results. However, we excluded people who used cancer services in 2014. Fourth, the variables for which data were available were limited—some data were unavailable in the NHI database, including on the history of cancer, cardiovascular disease, and other diseases, for those who did not use medical services or undergo health check-ups. Specific health check-ups do not target cancer diagnosis; instead focus on identifying conditions such as cardiovascular diseases. In addition, the number of insurance claims for outpatient medical services does not represent the use of personal primary care doctors. We could not notify the claims for outpatient and inpatient medical services, which were not related to cancer screening. A data management system which gathers the individual history of cancer screening and records of personal primary care doctors has not been established in Japan. Such a system could enhance future studies. Fifth, if individuals who use private health services, including cancer screening, are classified into the group abstaining from health check-ups, differential misclassification may occur. Misclassification may influence the association between the use of health check-ups and cancer incidence, but the direction of the effect would decrease the risk estimation. Hence, the risk from our results was acceptable. Moreover, it can be assumed that few NHI members are able to access expensive private preventive health services. Thus, we conclude that this issue did not significantly influence our findings. Sixth, cancer types were not considered individually in the analysis, although the actual preventive effect of screening varies with the cancer type. Not all cancers can be diagnosed through screening, for example, cancers of the head and neck region. Seventh, it was difficult to ascertain causality. The study was observational and had few variables. Thus, unmeasured confounders could be correlated with indicator variables and outcomes. Unmeasured confounders may include history of disease, smoking, alcohol consumption, and socio-economic status. However, knowledge about factors related to abstinence from health check-ups in Japan is limited. Further research is needed to confirm the confounding factors. Moreover, the follow-up period was relatively short. Finally, a larger sample size is required to conduct sufficient statistical tests for each cancer site (e.g., topology).

## Conclusions

This is the first study to investigate the relationship between undergoing specific health check-ups and cancer diagnosis, which is a critical target of preventive medicine health policies in Japan. Among NHI members, abstaining from health check-ups was a predictor of cancer diagnosis and advanced cancer. Reassessment of primary prevention strategies is required to investigate the baseline condition of NHI members who do not undergo health check-ups. Moreover, future research should identify secondary prevention strategies, which facilitate adequate preventive health service usage, through health education and recommendations from health professionals.

## Abbreviations

NHI: National Health Insurance
EHI: Employee’s Health Insurance
SEER: Surveillance Epidemiology and End Results

## Supplementary information

**Additional file 1:** Description of the method used to examine the accuracy of data matching.

**Additional file 2:** Number of cases of frequent cancers identified in this study.

**Additional file 3:** Baseline characteristics of participants according to health check-up participation.

**Additional file 4:** Association between predictors and diagnosis of either of the four most frequent cancers.

**Additional file 5:** Risk of diagnosing advanced-stage cancer among individuals who were diagnosed with either of the four most frequent cancers.

## Declarations

### Availability of data and materials

The datasets used and/or analysed during the current study are available from the corresponding author on reasonable request.
