# Supplementary material for: Abstaining from annual health check-ups is a predictor of advanced cancer diagnosis: a retrospective cohort study
Source: Environ Health Prev Med. 2022 Feb 19;27:1. doi: 10.1265/ehpm.21-00292 (PMC9093613; doi:10.1265/ehpm.21-00292)
Supplement: Supplementary file 1 — Additional file 1: Description of the method used to examine the accuracy of data matching. [file ehpm-27-001-s001.pptx]

## Slide 1
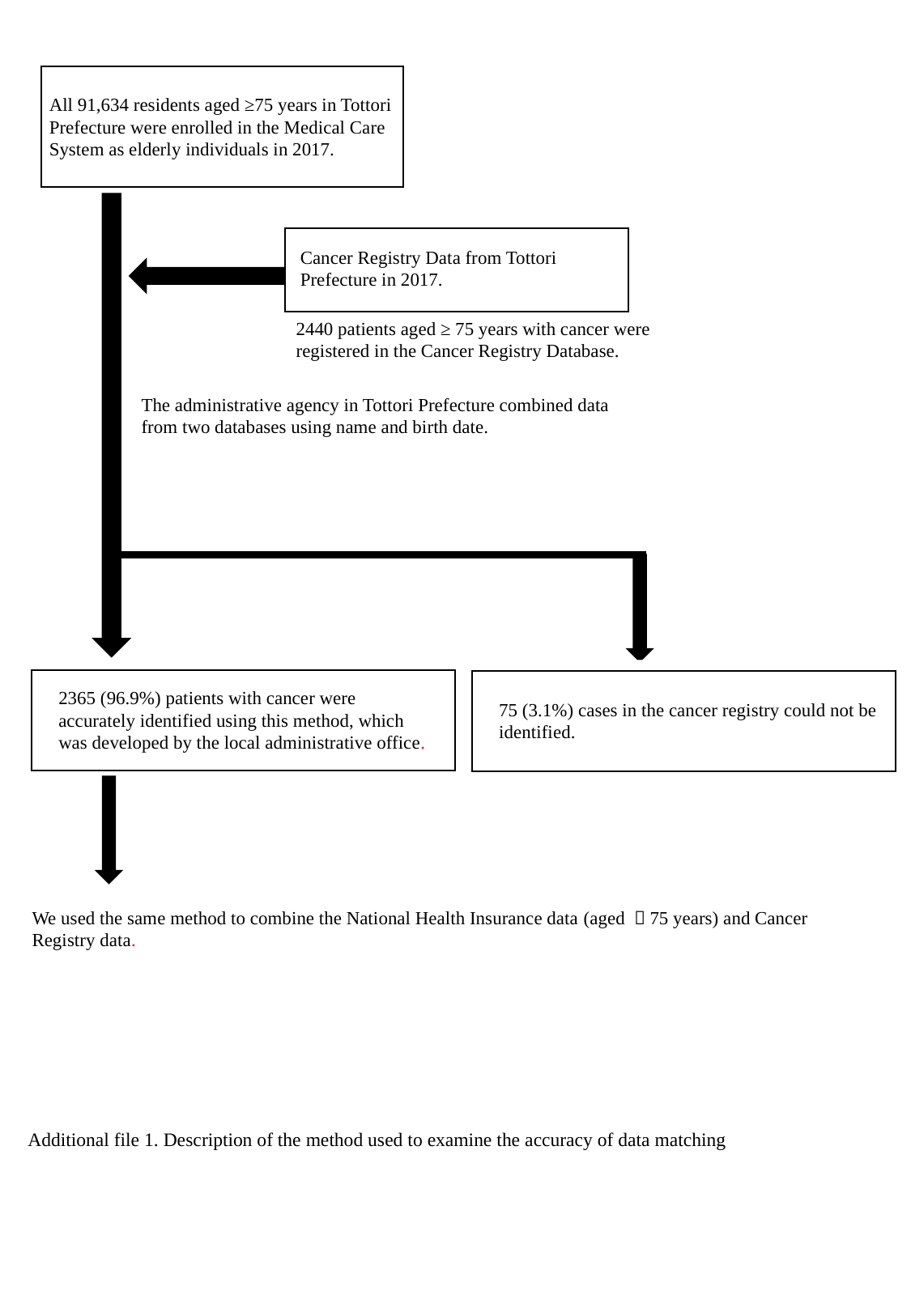

All 91,634 residents aged ≥75 years in Tottori Prefecture were enrolled in the Medical Care System as elderly individuals in 2017.
Cancer Registry Data from Tottori Prefecture in 2017.
2440 patients aged ≥ 75 years with cancer were registered in the Cancer Registry Database.
The administrative agency in Tottori Prefecture combined data from two databases using name and birth date.
2365 (96.9%) patients with cancer were accurately identified using this method, which was developed by the local administrative office.
75 (3.1%) cases in the cancer registry could not be identified.
We used the same method to combine the National Health Insurance data (aged ＜75 years) and Cancer Registry data.
Additional file 1. Description of the method used to examine the accuracy of data matching
