# Supplementary material for: Abstaining from annual health check-ups is a predictor of advanced cancer diagnosis: a retrospective cohort study
Source: Environ Health Prev Med. 2022 Feb 19;27:1. doi: 10.1265/ehpm.21-00292 (PMC9093613; doi:10.1265/ehpm.21-00292)
Supplement: Supplementary file 2 — Additional file 2: Number of cases of frequent cancers identified in this study. [file ehpm-27-001-s002.docx]

Additional file 2. Number of cases of frequent cancers identified in this study

| Cancer sites (ICD-O-3 codes) | | | Cancer stage (SEER summary staging manual 2000) | | | |
| --- | --- | --- | --- | --- | --- | --- |
|  |  |  | *In situ* or localised | Regional or distant | Unknown if extension or metastasis | Not applicable |
|  | n | (%) | n | n | n | n |
| All sites | 920 | (100.0) | 475 | 407 | 26 | 12 |
| Stomach (C16.x) | 123 | (13.4) | 77 | 44 | 2 | 0 |
| Colorectum (C18.x, C19.x, C20.x) | 204 | (22.2) | 102 | 97 | 5 | 0 |
| Lung (C34.x) | 120 | (13.0) | 43 | 75 | 2 | 0 |
| Liver (C22.x) | 36 | (3.9) | 27 | 9 | 0 | 0 |
| Breast (C50.x) | 83 | (9.0) | 54 | 26 | 3 | 0 |
| Uterus (C53.x, C54.x, C55.x) | 32 | (3.5) | 21 | 10 | 1 | 0 |
| Prostate (C61.x) | 61 | (6.6) | 36 | 23 | 2 | 0 |
| Thyroid (C73.9) | 19 | (2.1) | 9 | 10 | 0 | 0 |
| Pancreas (C25.x) | 28 | (3.0) | 1 | 27 | 0 | 0 |
| Others | 214 | (23.3) | 105 | 86 | 11 | 12 |
| ICD-O-3: International Classification of Diseases for Oncology Third Edition | | | | | | |
| SEER: The Surveillance Epidemiology and End Results | | | | | | |
