# Supplementary material for: Abstaining from annual health check-ups is a predictor of advanced cancer diagnosis: a retrospective cohort study
Source: Environ Health Prev Med. 2022 Feb 19;27:1. doi: 10.1265/ehpm.21-00292 (PMC9093613; doi:10.1265/ehpm.21-00292)
Supplement: Supplementary file 3 — Additional file 3: Baseline characteristics of participants according to health check-up participation. [file ehpm-27-001-s003.docx]

Additional file 3. Baseline characteristics of participants according to health check-up participation

|  |  | Participants who abstained from health check-ups in 2014 | | Participants who underwent health check-ups in 2014 | | Total | |
| --- | --- | --- | --- | --- | --- | --- | --- |
|  |  | n=52155, 72.3% | | n=20016, 27.7% | | n=72171 | |
|  |  | n | (%) | n | (%) | n | (%) |
| Sex |  |  |  |  |  |  |  |
|  | Female | 26278 | (50.4) | 12483 | (62.4) | 38761 | (53.7) |
|  | Male | 25877 | (49.6) | 7533 | (37.6) | 33410 | (46.3) |
| Age |  |  |  |  |  |  |  |
|  | 40–49 years | 7981 | (15.3) | 901 | (4.5) | 8882 | (12.3) |
|  | 50–59 years | 7982 | (15.3) | 1751 | (8.7) | 9733 | (13.5) |
|  | 60–69 years | 23430 | (44.9) | 9672 | (48.3) | 33102 | (45.9) |
|  | 70–74 years | 12762 | (24.5) | 7692 | (38.4) | 20454 | (28.3) |
| Residential area | |  |  |  |  |  |  |
|  | Urban | 32325 | (62.0) | 10311 | (51.5) | 42636 | (59.1) |
|  | Rural | 19830 | (38.0) | 9705 | (48.5) | 29535 | (40.9) |
| Number of months which insurance claim for outpatient medical service occurred in 2014 | | | | | | | |
|  | 0–1 | 20006 | (38.4) | 2918 | (14.6) | 22924 | (31.8) |
|  | 2–6 | 12160 | (23.3) | 5794 | (28.9) | 17954 | (24.9) |
|  | 7–10 | 7903 | (15.2) | 4315 | (21.6) | 12218 | (16.9) |
|  | 11–12 | 12086 | (23.2) | 6989 | (34.9) | 19075 | (26.4) |
| Insurance claims for Inpatient medical service in 2014 | | | | |  |  |  |
|  | None | 48398 | (92.8) | 18852 | (94.2) | 67250 | (93.2) |
|  | At least once | 3757 | (7.2) | 1164 | (5.8) | 4921 | (6.8) |
