# Supplementary material for: Abstaining from annual health check-ups is a predictor of advanced cancer diagnosis: a retrospective cohort study
Source: Environ Health Prev Med. 2022 Feb 19;27:1. doi: 10.1265/ehpm.21-00292 (PMC9093613; doi:10.1265/ehpm.21-00292)
Supplement: Supplementary file 4 — Additional file 4: Association between predictors and diagnosis of either of the four most frequent cancers. [file ehpm-27-001-s004.docx]

Additional file 4. Association between predictors and diagnosis of either of the four most frequent cancers

|  |  | Stomach cancer (123 cases) | | | | Colorectal cancer (204 cases) | | | | | | | Lung cancer  (120 cases) | | | | | | | | | Breast cancer  (82 cases) | | | | | | | | | | | |
| --- | --- | --- | --- | --- | --- | --- | --- | --- | --- | --- | --- | --- | --- | --- | --- | --- | --- | --- | --- | --- | --- | --- | --- | --- | --- | --- | --- | --- | --- | --- | --- | --- | --- |
|  |  | n=71374^a^ | | | | n=71455^b^ | | | | | | | n=71371^c^ | | | | | | | | | n (females only)=38453^e^ | | | | | | | | | | | |
|  |  | OR^*^ | (95% CI) | | | OR^*^ | | (95% CI) | | | | | OR^*^ | | | | (95% CI) | | | | | OR^*^ | | | (95% CI) | | | | | | | | |
| Health check-up in 2014 | | | | |  |  |  | | |  | |  | |  | |  | | |  | |  | |  | | |  | | | |  | |  |  |
|  | Received | 1.00 |  |  |  | 1.00 | |  |  | |  | | 1.00 | | | |  |  | |  | | 1.00 | | |  | | |  |  | | | | |
|  | Abstained | 1.10 | (0.73 | , | 1.65) | 1.57 | | (1.12 | , | | 2.21) | | 1.44 | | | | (0.93 | , | | 2.22) | | 0.73 | | | (0.46 | | | , | 1.18) | | | | |
| Number of months of insurance claims for outpatient medical services in 2014 | | | | | | | | | | | | | | | | | | | | | | | | | | | | | | | | | |
|  | 0–1 | 1.00 |  |  |  | 1.00 | |  |  | |  | | 1.00 | | | |  |  | |  | | 1.00 | | |  | | |  |  | | | | |
|  | 2–6 | 0.75 | (0.44 | , | 1.28) | 0.91 | | (0.61 | , | | 1.35) | | 0.79 | | | | (0.47 | , | | 1.34) | | 0.96 | | | (0.54 | | | , | 1.72) | | | | |
|  | 7–10 | 0.98 | (0.58 | , | 1.67) | 0.91 | | (0.59 | , | | 1.42) | | 0.83 | | | | (0.48 | , | | 1.45) | | 0.77 | | | (0.39 | | | , | 1.54) | | | | |
|  | 11–12 | 0.95 | (0.59 | , | 1.51) | 1.18 | | (0.82 | , | | 1.70) | | 0.92 | | | | (0.57 | , | | 1.48) | | 0.74 | | | (0.40 | | | , | 1.38) | | | | |
| Sex | |  |  |  |  |  | |  |  | |  | |  | | | |  |  | |  | |  | | |  | | |  |  | | | | |
|  | Female | 1.00 |  |  |  | 1.00 | |  |  | |  | | 1.00 | | | |  |  | |  | | NA | | |  | | |  |  | | | | |
|  | Male | 3.98 | (2.64 | , | 5.99) | 1.87 | | (1.41 | , | | 2.48) | | 2.77 | | | | (1.89 | , | | 4.08) | | NA | | |  | | |  |  | | | | |
| Age | | 1.12 | (1.08 | , | 1.16) | 1.09 | | (1.07 | , | | 1.12) | | 1.13 | | | | (1.09 | , | | 1.18) | | 1.00 | | | (0.98 | | | , | 1.03) | | | | |
| Residential area | |  |  |  |  |  | |  |  | |  | |  | | | |  |  | |  | |  | | |  | | |  |  | | | | |
|  | Urban | 1.00 |  |  |  | 1.00 | |  |  | |  | | 1.00 | | | |  |  | |  | | 1.00 | | |  | | |  |  | | | | |
|  | Rural | 1.25 | (0.87 | , | 1.78) | 0.65 | | (0.48 | , | | 0.88) | | 0.84 | | | | (0.58 | , | | 1.22) | | 0.82 | | | (0.52 | | | , | 1.30) | | | | |
| Insurance claims for in-patient medical services in 2014 | | | | | | | | | | | | |  | |  | |  | | |  | |  | |  | | |  | | | |  | |  |
|  | None | 1.00 |  |  |  | 1.00 | |  |  | |  | | 1.00 | | | |  |  | |  | | 1.00 | | |  | | |  |  | | | | |
|  | At least once | 0.77 | (0.37 | , | 1.59) | 0.86 | | (0.50 | , | | 1.49) | | 1.27 | | | | (0.69 | , | | 2.34) | | 1.35 | | | (0.58 | | | , | 3.14) | | | | |

* Adjusted for health check-up in 2014, number of months of insurance claims for outpatient medical services in 2014, sex, age, residential area, and insurance claims for in-patient medical services in 2014.

^a^ Participants who were diagnosed with cancers other than stomach cancer were excluded from the analysis.

^b^ Participants who were diagnosed with cancers other than colorectal cancer were excluded from the analysis.

^c^ Participants who were diagnosed with cancers other than lung cancer were excluded from the analysis.

^d^ Participants who were diagnosed with cancers other than breast cancer were excluded from the analysis.
