# Supplementary material for: Abstaining from annual health check-ups is a predictor of advanced cancer diagnosis: a retrospective cohort study
Source: Environ Health Prev Med. 2022 Feb 19;27:1. doi: 10.1265/ehpm.21-00292 (PMC9093613; doi:10.1265/ehpm.21-00292)
Supplement: Supplementary file 5 — Additional file 5: Risk of diagnosing advanced-stage cancer among individuals who were diagnosed with either of the four most frequent cancers. [file ehpm-27-001-s005.docx]

Additional file 5. Risk of diagnosing advanced-stage cancer among individuals who were diagnosed with either of the four most frequent cancers

|  |  | Stomach cancer (44 advanced cancers) | | | | Colorectal cancer  (97 advanced cancers) | | | | Lung cancer (75 advanced cancers) | | | | Breast cancer (26 advanced cancers) | | | |  |
| --- | --- | --- | --- | --- | --- | --- | --- | --- | --- | --- | --- | --- | --- | --- | --- | --- | --- | --- |
|  |  | n=121^a^ | | | | n=199^b^ | | | | n=118^c^ | | | | n (females only)=80^d^ | | | |  |
|  |  | OR^*^ | (95% CI) | | | OR^*^ | (95% CI) | | | OR^*^ | (95% CI) | | | OR^*^ | (95% CI) | | |  |
| Health check-up in 2014 | | | |  |  |  |  |  |  |  |  |  |  |  |  |  |  |  |
|  | Received | 1.00 |  |  |  | 1.00 |  |  |  | 1.00 |  |  |  | 1.00 |  |  |  |  |
|  | Abstained | 9.83 | (2.66 | , | 36.29) | 2.98 | (1.39 | , | 6.39) | 1.91 | (0.74 | , | 4.90) | 2.29 | (0.72 | , | 7.27) |  |
| Number of months of insurance claims for outpatient medical services in 2014 | | | | | | | | | | | | | | | | | | |
|  | 0–1 | 1.00 |  |  |  | 1.00 |  |  |  | 1.00 |  |  |  | 1.00 |  |  |  |  |
|  | 2–6 | 1.22 | (0.36 | , | 4.17) | 0.38 | (0.16 | , | 0.91) | 0.51 | (0.15 | , | 1.80) | 0.64 | (0.17 | , | 2.42) |  |
|  | 7–10 | 0.90 | (0.27 | , | 2.97) | 0.36 | (0.14 | , | 0.92) | 0.30 | (0.08 | , | 1.12) | 0.56 | (0.11 | , | 2.89) |  |
|  | 11–12 | 1.45 | (0.50 | , | 4.20) | 0.37 | (0.16 | , | 0.81) | 0.39 | (0.12 | , | 1.25) | 1.08 | (0.28 | , | 4.19) |  |
| Sex | |  |  |  |  |  |  |  |  |  |  |  |  |  |  |  |  |  |
|  | Female | 1.00 |  |  |  | 1.00 |  |  |  | 1.00 |  |  |  | NA |  |  |  |  |
|  | Male | 2.11 | (0.74 | , | 6.02) | 0.65 | (0.35 | , | 1.19) | 2.52 | (1.07 | , | 5.93) | NA |  |  |  |  |
| Age | | 0.99 | (0.91 | , | 1.09) | 1.01 | (0.95 | , | 1.07) | 0.95 | (0.85 | , | 1.05) | 0.97 | (0.90 | , | 1.04) |  |
| Residential area | |  |  |  |  |  |  |  |  |  |  |  |  |  |  |  |  |  |
|  | Urban | 1.00 |  |  |  | 1.00 |  |  |  | 1.00 |  |  |  | 1.00 |  |  |  |  |
|  | Rural | 1.01 | (0.43 | , | 2.39) | 1.38 | (0.72 | , | 2.62) | 0.49 | (0.21 | , | 1.16) | 1.75 | (0.60 | , | 5.11) |  |
| Insurance claims for in-patient medical services in 2014 | | | | | | | | | |  |  |  |  |  |  |  |  |  |
|  | None | 1.00 |  |  |  | 1.00 |  |  |  | 1.00 |  |  |  | 1.00 |  |  |  |  |
|  | At least once | 0.30 | (0.03 | , | 2.96) | 0.82 | (0.26 | , | 2.64) | 1.20 | (0.28 | , | 5.11) | 0.43 | (0.04 | , | 4.29) |  |

* Adjusted for health check-up in 2014, number of months of insurance claims for outpatient medical services in 2014, sex, age, residential area, and insurance claims for inpatient medical services in 2014.

^a^ Participants who were not diagnosed with stomach cancer were excluded from the analysis. Cancer cases that were categorised as ‘unknown’ or ‘unstaged’ were excluded.

^b^ Participants who were not diagnosed with colorectal cancer were excluded from the analysis. Cancer cases that were categorised as ‘unknown’ or ‘unstaged’ were excluded.

^c^ Participants who were not diagnosed with lung cancer were excluded from the analysis. Cancer cases that were categorised as ‘unknown’ or ‘unstaged’ were excluded.

^d^ Participants who were not diagnosed with breast cancer were excluded from the analysis. Cancer cases that were categorised as ‘unknown’ or ‘unstaged’ were excluded.
